# Steep uphill cycling using repeated transitions between seated and standing positions results in a lower blood-lactate concentration than continuous use of either seated or standing position

**DOI:** 10.3389/fspor.2024.1395415

**Published:** 2024-09-20

**Authors:** Magnus Carlsson, Oliver Lindblom, Tomas Carlsson

**Affiliations:** School of Health and Welfare, Dalarna University, Falun, Sweden

**Keywords:** bicycling, cycling posture, gross efficiency, treadmill, physiological response

## Abstract

This study investigated whether repeated transitions between seated and standing positions has a different physiological response compared to continuous use of either seated position or standing position during steep uphill cycling among elite cyclists. Ten elite male cyclists completed three 5-min treadmill cycling tests at an inclination of 6.8° with constant individual-based speed resulting in a work intensity close to the aerobic threshold. During the first and third test, the participants used standing position (ST test) and seated position (SE test) or vice versa, whereas in the second test, they made repeated transitions between standing and seated positions every 10 s (RT test). The last 2 min of each test was used to measure the mean values of oxygen uptake (V̇O_2_) and respiratory exchange ratio, which were used to calculate the metabolic rate (MR) and gross efficiency (GE). Additionally, the blood-lactate concentration before and after (La_post_) each test was determined. One-way repeated measures ANOVA was used to determine the effect of cycling position on the physiological response. No significant differences between tests were observed for the variables related to aerobic energy expenditure (i.e., V̇O_2_, MR and GE), whereas the RT test was associated with a significantly lower La_post_ compared to the ST and SE tests. Steep uphill cycling, at an intensity close to the aerobic threshold, with repeated transitions between standing and seated positions, did not have a higher oxygen consumption; instead, the blood-lactate concentration was lower during the RT test compared to that under continuous use of either seated or standing position.

## Introduction

In most endurance sports, such as cycling, it is important for athletes to be energy efficient by having a high gross efficiency (GE). In a review, it has been demonstrated that GE explained approximately 30% of the variation in power output (PO) during cycling time-trials (1). Hence, for a given PO a cyclist with a high GE will have a lower energy expenditure than a matched counterpart with lower GE. This performance advantage could be of great importance in a cycling race, where the cyclists must overcome force of gravity, rolling resistance, and air resistance. On flat terrain with cycling speeds above 10 m·s^−1^ (≈36 km·h^−1^), the contribution of aerodynamic drag to the resistive forces is over 90% (2). However, the air resistance could be reduced substantially by drafting behind other cyclists and thereby reducing the energy expenditure (3). When the road inclination increases, the cycling speed for a given power output is reduced (4), and the major resistive force is the force of gravity (5–7). In uphill segments, cyclists alternate between seated position and standing position to maintain a constant speed by adjusting the balance between pedaling cadence and tangential force (8). It was found that elite cyclists spent 22.4% of their time cycling in standing position during an uphill time-trial with a mean inclination of 4.0° (i.e., 7.0%) (8), whereas corresponding proportion of standing cycling was 34% at a gradient of 2.9° (i.e., 5.0%) and a power output equal to 93% of the power output associated with the work intensity when maximal oxygen consumption (V̇O_2_max) occurs (Ẇmax) (9).

Several studies have investigated similarities and differences in physiological response during uphill cycling in seated and standing positions. Oxygen consumption (V̇O_2_), have been found to be lower for cycling in seated position than standing position for a variety of inclinations from 2.3° to 5.7° (i.e., 4.0%–10.0%) (10–14), but there are also studies that found no difference in V̇O_2_ between these two positions for the same range of inclinations (8, 9, 11, 12, 15). These contradicting findings are also present for different measures of energy efficiency, where both no difference between seated and standing cycling (9, 16) and higher efficiency for seated position (11, 13) have been found. The blood-lactate concentration did not differ between seated and standing positions during submaximal treadmill cycling at an inclination of 5.7° (i.e., 10.0%) (11, 15) or outdoor cycling at a mean inclination of 2.9° and different submaximal work intensities (9).

Cycling performance at a fixed grade of 5.7° was found to be better for standing position than seated position at power outputs exceeding 94% of Ẇmax (15). Previous studies reported that the spontaneous positional change from seated to standing position with increasing power output is done to minimize muscular efforts (17, 18).

In a review article, it was pointed out that the magnitude and activity of several key muscle groups differ between cycling in seated and standing positions (19). Differences in activation between these two positions have been found for lower-body muscles such as gluteus maximus, rectus femoris, biceps femoris, vastus medialis, soleus, tibialis anterior, m. semimembranosus, and gastrocnemius (13, 14, 20, 21). There are also muscle-activation differences in the upper body (e.g., biceps brachii, rectus abdominis, latissimus dorsi, and erector spinae) between these two positions (21, 22), and the work done by the upper limbs is reported to be greater in standing position (23).

Recently, we showed that repeated sub-technique transitions, between diagonal-stride technique (DS) and double-poling technique (DP) every 6 s during treadmill roller skiing at an inclination of 2.5°, was not associated with a reduced GE compared to that under continuous use of DS and DP (24). However, the pre-post difference in blood-lactate concentration was significantly lower for the test with repeated sub-technique transitions between DS and DP. One potential contributing factor to the lower blood-lactate concentration, when the sub-techniques are alternated, is the frequent unloading of the working muscles in each sub-technique which leads to a better oxygenation and thereby a lower blood-lactate production and/or a better blood-lactate clearance.

Based on the reasoning above related to sub-technique transitions in cross-country skiing, cyclists' shift between seated and standing positions aiming to minimize muscular efforts, and the reported muscle-activation differences between cycling in seated and standing positions, it would be of great interest to examine the physiological response of frequent shifts between these two cycling positions using a work intensity that could be sustained during continuous steep uphill cycling. No previous study has investigated if the aerobic energy contribution and blood-lactate concentration post exercise differ between standing cycling, seated cycling, and cycling using repeated shifts between these positions at an inclination suitable for both seated and standing positions. Therefore, the purpose of this study was to investigate whether repeated transitions between seated and standing positions has a different physiological response compared to continuous use of either seated position or standing position during steep uphill cycling among elite cyclists.

## Materials and methods

### Participants

Ten elite male cyclists (age: 25 ± 8 years; stature: 1.78 ± 0.07 m; body mass: 75.9 ± 8.4 kg) volunteered to participate in the study. All cyclists competed at a high national level, have top-20 placement at the Swedish National Championships, and six of them have at least one podium place. All subjects gave their written informed consent to participate in the study. The test procedures were performed in accordance with the World Medical Association's Declaration of Helsinki—Ethical Principles for Medical Research Involving Human Subjects 2008, and the study was approved by the Swedish Ethical Review Authority (Dnr 2022-01504-01).

### Testing procedures

The participants were instructed to perform only light training on the two days preceding their scheduled test day, to be well hydrated, to refrain from alcohol (24 h) and caffeine (12 h) and to avoid eating within 2 h prior to testing. On the day of the tests, the participants completed a health-status questionnaire, and thereafter, each participant's stature (Harpenden Stadiometer, Holtain Limited, Crymych, Great Britain) as well as body mass and mass of the equipment (i.e., bike, shoes, safety harness, and helmet) (Midrics 2, Sartorius AG, Goettingen, Germany) were measured.

The warm-up started with 5 min cycling on a motor-driven treadmill (Saturn, 450/300rs, h/p/cosmos sports & medical GmbH, Nussdorf-Traunstein, Germany) at an inclination of 2.5° and speed of 4.2 m·s^−1^ (i.e., 15 km·h^−1^). Thereafter, the rolling-resistance coefficient (*μ*) of the participant's own bicycle was determined using a previously described method (25). In brief, the treadmill speed was set at 5.6 m·s^−1^ (i.e., 20 km·h^−1^), with the rider facing downhill, and the treadmill's negative inclination was then adjusted until the participant sitting on the bicycle (without pedaling) did not move in either the backward or forward direction on the treadmill. Based on the equilibrium inclination, the μ was calculated from the formula (*m*_tot_ · *g* · sin *α*)/(*m*_tot_ · *g* · cos *α*), where *m*_tot_ is the total mass of the participant and equipment (kg), *g* is the acceleration due to gravity (9.82 m·s^−2^ at the location of the sport-science laboratory) and *α* is the treadmill inclination (°).

To determine an adequate and individual-based work intensity for the three cycling tests, the participants performed three 6-min submaximal stages of 2.5, 3.0, and 3.5 W·kg^−1^ with 2 min of rest between stages. During the rest periods, capillary blood samples were collected from a fingertip and thereafter analyzed to determine blood-lactate concentrations (mmol·L^−1^) (Biosen 5140, EKF-diagnostic GmbH, Barleben, Germany). Based on the relationship between work intensity and blood-lactate concentration, an individual-based treadmill speed was determined to correspond to a work intensity close to the aerobic threshold (i.e., a blood-lactate concentration of 2 mmol·L^−1^). During the last 3 min of the warm-up, the pre-determined treadmill speed/inclination-combination (i.e., the individual-based work intensity) was tested and the participants selected a suitable gear for seated position and standing position, respectively, which were used during the 5-min tests using standing position (ST test) and seated position (SE test). The participants were also familiarized with the alteration between seated and standing positions, which was used in the repeated transition test (RT test).

**Figure 1:**
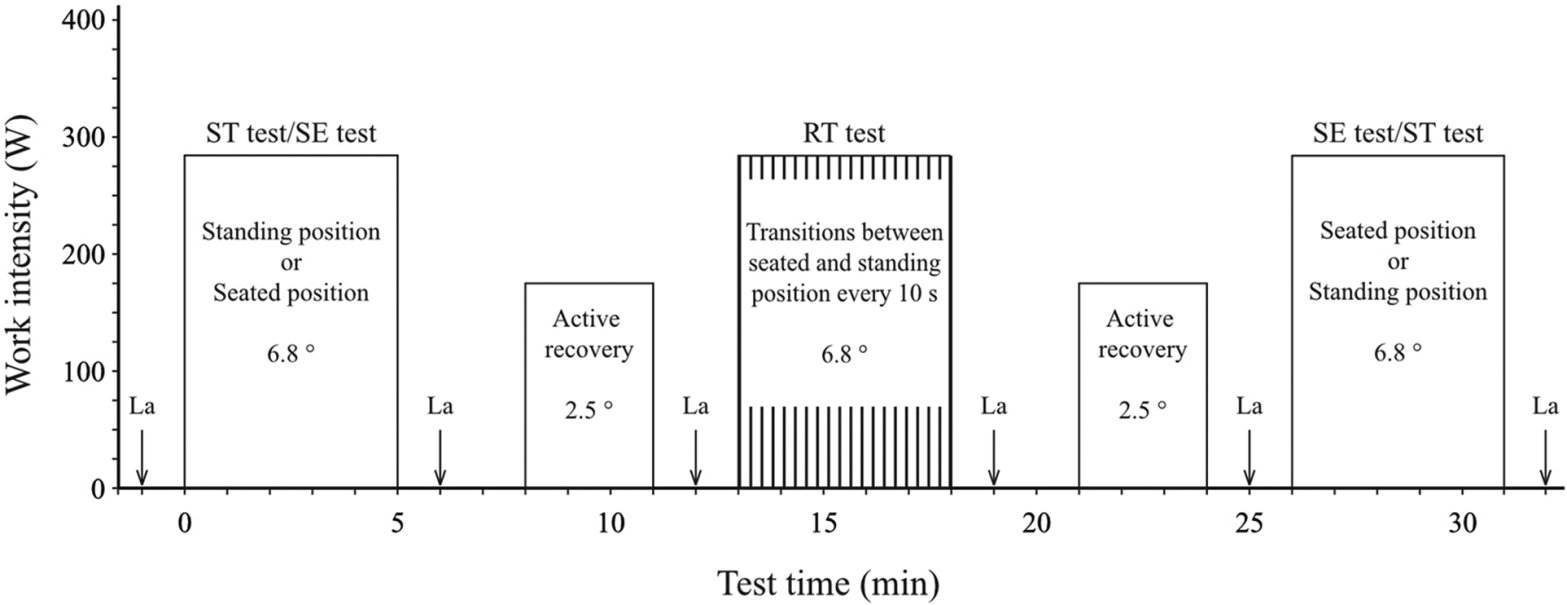
Overview of the test procedure for the 5-min submaximal cycling tests, where ST is standing position, SE is seated position, RT is repeated transitions between standing and seated positions, and La is blood-lactate sampling.

Thereafter, the three cycling tests were initiated using a randomized counterbalanced crossover design (Figure 1). The participants randomized to the first group followed the test order SE test – RT test – ST test, whereas the test order for the second group was ST test – RT test – SE test. In each test, the participants cycled 5 min at a treadmill inclination of 6.8° while using their individual set treadmill speed. During the ST and SE tests, the participants used the pre-determined cycling position throughout the test, whereas in the RT test they made transitions between seated and standing positions every 10 s. Each transition was preceded by a 3-s countdown (Stopwatch, Fitlb, San Jose, USA) with one beep per second followed by a higher tone that was regarded as the intended time of the alteration of the cycling position.

The rest period between tests was 8 min. Within 1 min after the test was completed a capillary blood sample (La_post_) was collected, which was followed by a new measurement of the mass of the participants to enable adjustment of the PO calculation for the subsequent test. Thereafter, the participants cycled at a low work intensity (*α* = 2.5° and *v* = 4.2 m·s^−1^) for 3 min. Within 1 min before the next test, another capillary blood sample (La_pre_) was collected.

Throughout the tests, the heart rate (HR) was monitored using a heart-rate sensor (Polar HR10, Polar Electro Oy, Kempele, Finland) and expired air was continuously analysed using a metabolic cart in mixing-chamber mode (Jaeger Oxycon Pro, Erich Jaeger Gmbh, Hoechberg, Germany). The last 2 min of each 5-min test was used to determine the mean values of V̇O_2_ (L·min^−1^) and respiratory exchange ratio (RER) (L·L^−1^). The metabolic rate (MR) (J·s^−1^) was calculated using the formula (3.815 + 1.232 · RER) · V̇O_2_ · *k*_1_, where *k*_1_ is 69.73 and converts kcal·min^−1^ to J·s^−1^ (i.e., W). The PO (W) is the sum of the work against gravity and the work related to overcoming the rolling resistance of the cycle; the PO was calculated in accordance with the formula (*m_tot_* · *g* · sin *α* · *v* + *m_tot_* · *g* · cos *α* · *μ* · *v*), where *v* is the treadmill speed (m·s^−1^). The GE is the ratio of the PO to MR.

### Statistical analyses

Test results are presented as the means and standard deviations. The homogeneity of the variances of the test variables was tested using Levene's test. The normality of the distributions of test variables was assessed by using the Shapiro–Wilk test. To determine the effect of cycling position on the physiological response, one-way repeated measures analysis of variance (ANOVA) was used. If the assumption of sphericity is violated, the results from Greenhouse-Geisser test is reported. Student's paired samples *t*-test was used as *post hoc* test to investigate test-variable differences between the ST, RT and SE tests. Cohen's effect-size criteria were used to interpret the magnitude of the effect size (*η*^2^) and to enable more informative inferences to be made from the results. The substantial effects were divided into more finely graded magnitude ranges as follows: small effect for 0.01 ≤ *η*^2^ < 0.06, moderate effect for 0.06 ≤ *η*^2 ^< 0.14, and large effect for *η*^2^ ≥ 0.14 (26). All statistical analyses were assumed to be significant at an alpha level of 0.05. The statistical analyses were conducted using IBM SPSS Statistics software, Version 28 (IBM Corporation, Armonk, USA).

## Results

Test results are presented in Table 1. The ANOVA tests showed no significant differences between tests for the variables *v*, *α*, *μ*, *m*_tot_, PO, V̇O_2_, MR, GE, and La_pre_ (Table 1). However, significant between-test differences were found for the variables RER and La_post_ (Table 1).

**Table 1:** Test results for the three cycling tests.

Variable	SE	RT	ST	*F*	*P*	*η* ^2^
*v* (m·s^−1^)	2.63 ± 0.30	2.63 ± 0.30	2.63 ± 0.30	0.00	1.00	0.00
*α* (°)	6.8 ± 0.0	6.8 ± 0.0	6.8 ± 0.0	0.00	1.00	0.00
μ (N·N^−1^)	0.0062 ± 0.0014	0.0062 ± 0.0014	0.0062 ± 0.0014	0.00	1.00	0.00
*m*_tot_ (kg)	88.5 ± 8.6	88.6 ± 8.6	88.6 ± 8.6	0.20	0.69	0.021*
PO (W)	284 ± 39	284 ± 39	284 ± 39	0.14	0.74	0.015*
V̇O_2_ (L·min^−1^)	3.86 ± 0.45	3.94 ± 0.45	3.86 ± 0.42	2.80	0.12	0.24*
RER (L·L^−1^)	0.92 ± 0.03^b^	0.90 ± 0.03^a,c^	0.92 ± 0.03^b^	8.06	0.0032	0.47
MR (J·s^−1^)	1,331 ± 149	1,352 ± 147	1,331 ± 139	1.56	0.24	0.15
GE (%)	21.3 ± 1.2	21.0 ± 1.1	21.3 ± 1.1	1.69	0.21	0.16
La_pre_ (mmol·L^−1^)	1.4 ± 0.5	1.2 ± 0.4	1.3 ± 0.7	0.51	0.51	0.053*
La_post_ (mmol·L^−1^)	2.2 ± 0.6^b^	1.8 ± 0.6^a,c^	2.5 ± 0.7^b^	5.43	0.014	0.38

Test results are presented as mean ± standard deviation. SE, seated cycling; RT, repeated transitions between seated and standing cycling; ST, standing cycling; *v*, treadmill speed; α, treadmill inclination; μ, rolling resistance of the bicycle; *m*_tot_, mass of the participant, including the mass of the equipment; PO, power output; V̇O_2_, oxygen consumption; RER, respiratory exchange ratio; MR, metabolic rate; GE, gross efficiency; La_pre_, blood-lactate concentration before the test; La_post_, blood-lactate concentration after the test. Results from the one-way repeated measures ANOVA is presented as *F*-, *P*-, and *η*^2^-values. Results from the post-hoc test are reported as follows:.

* Indicates that results from the Greenhouse-Geisser test is reported.

^a^ Represents significant (*p* < 0.05) difference to the SE test.

^b^ Represents significant difference to the RT test.

^c^ Represents significant difference to the ST test.

**Figure 2:**
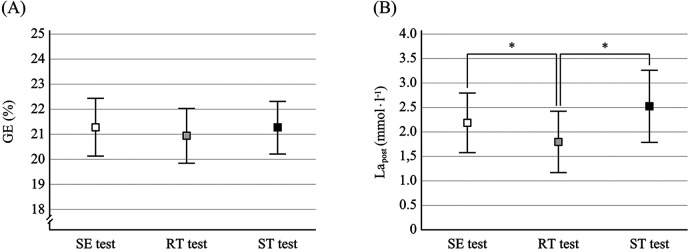
The effect of cycling position on **(A)** gross efficiency (GE) and **(B)** blood-lactate concentration after the test (la_post_), during 5-min cycling tests using seated position (SE test), standing position (ST test), and repeated transitions between seated and standing positions (RT test). Significant difference between tests is reported as *P < 0.05. Squares represent mean values and error bars represent ± 1 standard deviation.

The *post hoc* analyses showed that RER was significantly lower after the RT test compared to both the SE test (*t* = −2.61; *p* = 0,014; *η*^2^ = 0.43) and the ST test (*t* = −5.93; *p* < 0.001; *η*^2^ = 0.80), whereas no difference was found between the SE test and the ST test (*t* = −0.80; *p* = 0.22; *η*^2^ = 0.066). For the variable La_post_, a significantly lower blood-lactate concentration was found for the RT test compared to both the SE test (*t* = −2.80; *p* = 0,021; *η*^2^ = 0.47) and the ST test (*t* = −3.01; *p* = 0,015; *η*^2^ = 0.50) (Figure 2). However, no difference in La_post_ was found between the SE test and the ST test (*t* = −1.29; *p* = 0.11; *η*^2^ = 0.16).

## Discussion

The results of this study demonstrate that during treadmill cycling at an inclination of 6.8°, corresponding to a work intensity close to the aerobic threshold, repeated transitions between seated and standing positions did not differ in oxygen consumption compared to cycling with continuous use of seated position or standing position. However, the blood-lactate concentration after the RT test was significantly lower than the corresponding concentrations after the SE and ST tests.

Previously, some studies have reported a lower V̇O_2_ and/or higher energy efficiency for seated position than standing position in gradients between 2.3° and 5.7° and at different work intensities (10–14), whereas other studies found no significant difference between positions for these variables at the same range of inclinations and work intensities (8, 9, 11, 12, 15, 16). In the current study, neither V̇O_2_ nor GE differed between the SE and ST tests, which partly could be explained by the greater treadmill inclination compared to the gradient use in the other studies, because standing cycling is gradually more favoured when inclination increases (7, 12). Based on the similarity between the SE and ST tests regarding the MR, it would be expected that the RT test, consisting of an equal proportion of seated and standing cycling, is associated with a higher V̇O_2_ and thereby a lower GE because of the additional work against gravity the cyclists performed when they change to standing position every 20 s. However, the marginally elevated V̇O_2_ of less than 0.1 L·min^−1^ did not result in significant difference between the tests for either V̇O_2_ or GE (Table 1).

A novel finding in the current study was the significantly lower La_post_ for the RT test compared to the SE and ST tests, which is consistent with the findings for cross-country skiers where repeated changes between DS and DP resulted in a lower La_post_ compared to continuous use of the sub-techniques (24). The repeated transitions between seated and standing position entail an involvement of a larger muscle mass than continuous use of either position, which is reflected by differences in muscle activation between cycling positions (20, 21). Hence, the alteration of the force-velocity and force-length relationships of power producing muscles when shifting cycling position (21), would lead to a lower activity of the muscles that are active during seated cycling when the cyclist uses standing position or vice versa. During each 10-s period of reduced muscle activity, there is an opportunity to for example resynthesize phosphocreatine in the “inactive” musculature that can be used during the subsequent 10-s period of increased activity. This would lead to a reduced anaerobic energy contribution from glycolytic processes during the RT test compared to the other two tests, which would contribute to the lower La_post_. Hence, the use of a lower muscle mass during continuous cycling in seated or standing position will put a higher emphasis on anaerobic energy contribution in the principally active muscles to meet the power production demand. In addition, the lower La_post_ could partly also be reflected by a better blood-lactate clearance and it is likely that these two factors contribute to the reduced blood-lactate concentration after the RT test.

It has been reported that elite cyclists during a 140-min steady state work, corresponding to a blood-lactate concentration of approximately 1.8 mmol·L^−1^, reduced their muscle glycogen content in vastus lateralis by 65% (27). A gradual glycogen depletion of individual muscle fibres has been suggested to be an essential factor explaining fatigue development and thereby an impaired performance (28). Based on the results in the current study, it appears that repeated transitions between seated and standing positions during prolonged submaximal cycling in steep uphill sections could postpone fatigue in the working muscles by reducing the metabolic load; a positive effect that was proposed 15 years ago by two different research groups (15, 21). Hence, it could be recommended for elite cyclists to adopt a strategy for positional changes during steep uphill cycling, at a work intensity close to the aerobic threshold, to reduce the blood-lactate concentration and eventually improve performance. However, future studies are needed to further investigate if a positive effect on metabolic load is present for work intensities above the intensity of approximately 1.8–2.5 mmol·L^−1^ that was used in the current study. It is also of great importance to investigate the ecological validity by doing the cycling tests outdoor using a fixed speed at an incline close to 6.8°. In addition, it would be of interest to determine the minimum and maximum inclines where repeated transitions between seated and standing positions could be beneficial from a metabolic perspective as well as the optimum timing of the positional changes.

## Conclusions

Steep uphill cycling, at a work intensity close to the aerobic threshold, with repeated transitions between seated and standing positions, did not differ in oxygen consumption compared to that under continuous use of either cycling position among elite male cyclists. Instead, uphill cycling with repeated sub-technique transitions resulted in a lower post test blood-lactate concentration.

## Data Availability

The raw data supporting the conclusions of this article will be made available by the authors, without undue reservation.
